# Shotgun metagenomic analysis of the tongue-coating microbiome reveals oral microbes and their functions in older adults with dementia

**DOI:** 10.1080/20002297.2026.2643036

**Published:** 2026-03-11

**Authors:** Jun Hyung Cha, Sol-Ah Jeong, Byoung-Seok Ye, Insuk Lee, Bock-Young Jung

**Affiliations:** a Department of Biotechnology, College of Life Science and Biotechnology, Yonsei University, Seoul, Republic of Korea; b Department of Advanced General Dentistry, College of Dentistry, Yonsei University, Seoul, Republic of Korea; c Department of Neurology, Yonsei University College of Medicine, Seoul, Republic of Korea; d DECODEBIOME Co., Ltd., Incheon, Republic of Korea

**Keywords:** Dementia, oral microbiome, shotgun metagenomic sequencing, biomarker, metabolic pathway

## Abstract

**Introduction:**

Dementia poses a growing burden in the aging population, prompting the search for noninvasive biomarkers for early detection.

**Materials and methods:**

We performed shotgun metagenomic sequencing of tongue-coating samples from older adults with dementia (*n *= 30) and cognitively healthy controls (*n *= 28) to identify oral microbiome signatures.

**Results:**

The analysis revealed distinct microbial compositions associated with dementia, including an enrichment of *Veillonella parvula* in dementia patients, whereas *Lautropia dentalis* was more abundant in healthy controls. We also identified functional alterations in the microbiome in the dementia group, including increased abundance of the histidine degradation and biotin biosynthesis pathways, whereas ubiquinol biosynthesis was more abundant in the healthy control group. The abundance of several microbial taxa and metabolic pathways were correlated with scores on the Korean Mini-Mental State Examination 2nd edition (K-MMSE), a clinical assessment of dementia severity. *Prevotella pleuritidis, Actinomyces* sp., *Leptotrichia buccalis*, and *Leptotrichia* sp. were positively correlated, whereas *Oribacterium parvum* was negatively associated with K-MMSE scores. Among the metabolic pathways, glutamine/glutamate biosynthesis was positively correlated with cognitive performance.

**Conclusions:**

These results suggest that specific oral taxa and their metabolic functions are associated with cognitive status and may reflect underlying neurodegenerative processes.

## Introduction

Dementia is a syndrome characterized by a chronic progressive decline in cognitive function through some degree of neurological degeneration, which involves the progressive degeneration of cells in both the central nervous system (CNS) and peripheral nervous system [1–4]. The most common neurodegenerative diseases among the aging population are Alzheimer's disease (AD) and Parkinson's disease (PD) [3,5,6]. AD, accounting for approximately 60–70% of all dementia cases, manifests primarily as confusion and cognitive impairment due to the loss of neurons in the hippocampus, basal forebrain, and other cortical areas. A pathophysiological hallmark of AD is amyloid plaques and neurofibrillary tangles of the tau protein, both of which lead to neuronal cell death [1,7–11]. PD primarily results from the degeneration of dopaminergic and serotoninergic neurons, leading to motor dysfunction and cognitive impairments [1]. The pathological hallmark of PD is the presence of Lewy bodies caused by the abnormal accumulation of alpha-synuclein protein in nerve cells [1,12–14].

The definitive diagnosis of neurodegenerative brain diseases such as AD and PD can be confirmed only through an autopsy of brain tissue. However, since this is not feasible during life, combined analysis of multiple biomarkers and clinical features is required for diagnosis. Methods currently include analysis of proteins in cerebrospinal fluid (CSF), functional magnetic resonance imaging (MRI), and positron emission tomography (PET) with tracer molecules [3,9,15–17]. These techniques can be invasive, uncomfortable, time-consuming and expensive [1,3,18]. Given that neurodegenerative diseases are characterized by a prolonged presymptomatic phase during which neuronal degeneration occurs before the onset of clinical symptoms [3,4,10,19], early diagnosis and monitoring are essential for effective treatment and improving patient outcomes. In response, current research focuses on the development of noninvasive diagnostic tools to identify biomarkers associated with neurodegenerative diseases [1,3].

The oral microbiome is defined as the collective genome and gene products of all microorganisms, including bacteria, archaea, fungi, protozoa, and viruses, in the oral cavity [20–22]. The oral cavity harbors the second largest microbial community in the human body after the gut and serves as a rich source of specimens for predicting diverse protein functions through metagenomic analysis [10,23,24]. Dysbiosis of the oral microbiota, which manifests as biofilms throughout the oral cavity, may seriously impact health by altering bacterial community function. Numerous recent studies on oral health and neurodegenerative disease have revealed that oral microbiota can enter the brain through the nervous system, circulating blood, or the blood‒brain barrier [8,10,11,25,26].

The oral cavity represents a valuable source for the noninvasive collection of biospecimens, including cells, saliva, the microbiome, proteins, lipids, and metabolites present in exhaled breath [27]. The oral microbiome, which is easily detectable through saliva or tongue swab samples, offers significant advantages in terms of safety, ease of collection, and noninvasiveness. These characteristics make oral samples particularly promising for diagnostic applications and may also improve prognostic assessments [1,3,28,29].

The tongue coating, a major source of oral microbial populations, provides high sample stability because biofilms formed by tongue epithelial cells and microbiota are shed at a steady rate. Given its diverse oral niches and sensitivity to disease, the tongue-coating microbiota constitutes a valuable site for investigating oral microbial dynamics [30,31].

Next-generation sequencing (NGS) has led to significant advances in research on the human microbiome by providing high-throughput, high-resolution insights into microbial structure and function. Shotgun metagenomic sequencing provides an unbiased, comprehensive view of microbial diversity, enabling functional analysis and offering key insights into complex microbial interactions and their roles in disease processes [32–37].

However, despite progress in clinical‒pathological correlations, the role of the oral microbiome in neurodegenerative diseases such as dementia remains unclear, with limited research on older adults. The aim of this study is to analyze the oral microbiome of dementia patients and healthy older adults via shotgun metagenomics, investigating the potential of the oral microbiome as a biomarker for dementia diagnosis and prevention.

## Materials and methods

### Participant recruitment

This study recruited participants from Yonsei University Severance Hospital's Neurology Department and Yonsei University Dental Hospital's Advanced General Dentistry Department between March 2023 and May 2024. A total of 58 older adults (aged 65 years and above), including 30 with dementia and 28 cognitively healthy individuals, participated. Diagnoses were made by a neurologist using the K-MMSE, Clinical Dementia Rating Scale (CDR), Seoul Neuropsychological Screening Battery-II (SNSB), and brain MRI. To ensure reliable comparisons with dementia patients, cognitively healthy individuals were thoroughly evaluated by a neurologist using the K-MMSE, CDR, SNSB, and brain MRI and were selected as the control group.

The K-MMSE assessed orientation, memory, and language, whereas the CDR measured dementia severity in six areas, including memory, judgment, and personal care. The SNSB, a comprehensive neuropsychological test, was used to evaluate attention, language, visuospatial function, memory, and executive function. Brain MRI was used to identify structural abnormalities affecting cognition [38,39].

Individuals who met any of the following criteria were excluded from the study: (1) those who used antibiotics, antifungals, steroids, immunosuppressants, or investigational drugs within the previous month; (2) those who had long-term oral diseases (other than periodontal disease or dental caries), or were receiving ongoing dental treatment; or (3) those who had conditions impairing salivary secretion; (4) those who wore removable partial or complete dentures; or (5) those who had uncontrolled systemic diseases. The sample size was calculated using G*Power software (v3.1.9.4) to ensure a significance level of 0.05 and a power of 0.8, requiring at least 26 participants per group. To account for a 10% dropout rate, 30 participants with dementia and 28 cognitively healthy individuals were recruited. Written informed consent was obtained from all participants or their legal guardians. The study protocol was approved by the Institutional Review Board of Yonsei University Dental Hospital (IRB No. 2-2022-0034) and conducted in accordance with the Declaration of Helsinki.

### Sample collection

The participants were instructed to refrain from eating or drinking for at least one hour before tongue-coating sample collection. The samples were obtained using a sterile swab and preservative solution (Cat. No. ACN 21.01). The swab was used to collect the tongue-coating sample evenly from the dorsum of the tongue for 20 seconds, avoiding contact with the teeth. Samples with insufficient quantity or poor-quality extracted genomic DNA were excluded from the analyses.

### DNA extraction

DNA extraction was performed with the Soil Pro DNA Extraction Kit, following the manufacturer’s protocol. First, 0.25 g of sample was added to a PowerBead Pro Tube containing 800 µl of Cell Disruption Solution 1 (Solution CD1). The mixture was then vortexed at maximum speed for 10 minutes to ensure thorough homogenization. After vortexing, the sample was centrifuged at 15,000 × g for 1 minute, and the supernatant was carefully transferred to a clean tube. The inhibitors were removed, and the DNA was purified using Solutions CD2, CD3, EA, and C5 in sequence. Finally, the purified DNA was eluted using Solution C6.

### Shotgun metagenomic sequencing

The sequencing libraries were prepared using the TruSeq Nano DNA High-Throughput Library Prep Kit (Illumina), following the manufacturer's instructions. The genomic DNA (100 ng) was quantified using the Qubit system to ensure high quality (260/280 ratio: 1.8–2.0). The DNA was fragmented to ~350 bp using Covaris acoustic technology. End repair was performed to create 5'-phosphorylated, blunt-ended DNA molecules, followed by size selection using a bead-based method to retain ~470 bp fragments.

A-tailing and ligation with indexed adapters subsequently enabled multiplex sequencing. Ligation products were purified and enriched through 8-cycle polymerase chain reaction (PCR) to amplify adapter-ligated fragments. The libraries were quantified via quantitative PCR (qPCR) and analyzed on an Agilent TapeStation, confirming an average insert size of ~470 bp for the 350 bp target design. Sequencing on the Illumina NovaSeq platform (2×150 bp) generated ~6 Gb of data per sample with a Q30 score >85%, ensuring high fidelity. FASTQ files were demultiplexed and processed using bcl2fastq to remove adapters and low-quality bases, preparing them for metagenomic analysis. Raw data from Illumina HiSeq (Illumina's high-throughput sequencing system) and NovaSeq were demultiplexed by index sequences to generate paired-end FASTQ files.

### Bioinformatics and data analysis

Adapter sequences and reads with an average Phred score less than 20 were removed using the Kneaddata (v0.10.0) [40] pipeline with Trimmomatic (v0.39, SLIDINGWINDOW:4:20) [41]. Reads aligning to the human genome (GRCh37) were further eliminated using Bowtie2 (v2.4.5) within Kneaddata. The microbial species profile was then generated from the preprocessed data using MetaPhlAn4 (v4.0.0) [42].

The Shannon and Simpson diversity indices were calculated from the species profiles by using the ‘calculate_diversity. R’ script in MetaPhlAn4. For beta diversity, differences in species composition between samples were assessed using the Bray‒Curtis distance, and principal coordinate analysis (PCoA) was performed using the R package *vegan* [43] to visualize these differences. In addition, we performed principal component analysis (PCA) on centered log-ratio (CLR)-transformed relative abundances using the Aitchison distance with the R package compositions [44].

Differentially abundant species between the dementia and healthy control groups were identified using MaAsLin2 [45], adjusting for potential confounding factors such as age, BMI, and sex. Statistically significant differences were determined based on *q*-values (adjusted *p*-values) calculated using Benjamini‒Hochberg false discovery rate (BH-FDR) correction for multiple comparisons. In addition, we repeated the MaAsLin2 analyses after CLR transformation of relative abundances.

Metabolic pathway abundance profiles were generated using the Human Microbiome Project Unified Metabolic Analysis Network 3 (HUMAnN3) (v 3.5) (40) based on the MetaCyc pathway database.

All analysis steps were performed with version-controlled tools and reproducible pipelines to ensure the consistency and reliability of the results.

### Statistical analysis

Statistical analyses were conducted using R (v4.4.1). The normality of continuous variables was assessed with the Shapiro‒Wilk test. Group differences in continuous variables were analyzed using the Student's t-test or the Wilcoxon rank-sum test, whereas categorical variables were tested with the chi-squared test.

Relative abundances and *α* diversity were compared between the dementia and healthy control groups by using the Wilcoxon rank-sum test, with *p* < 0.05 considered significant. Beta diversity differences in microbial community composition were assessed using PERMANOVA (adonis2, vegan package). Differential abundance analysis of microbial species and pathways was performed by applying multivariate linear models with MaAsLin2. Dementia/healthy status was treated as a fixed effect, whereas age, BMI, and sex were included as random effects to adjust for confounders. Statistical significance was determined using a *q*-value (FDR-adjusted *p*-value) cutoff of 0.05 for multiple comparisons.

## Results

### Dementia patients display a distinctive oral microbiota composition

The mean age of all participants was 77.48 years, ranging from 65 to 86 years. The average age of the dementia group was significantly higher than that of the healthy control group (*p*-value = 0.0005). The Korean Mini-Mental State Examination 2nd edition (K-MMSE) score was significantly lower in the dementia group (19.23 ± 3.86) than in the healthy control group (28.36 ± 1.22) (*p*-value < 0.0001). Meanwhile, body mass index (BMI) between the two groups show no difference (Supplementary Table 1).

**Figure 1.**
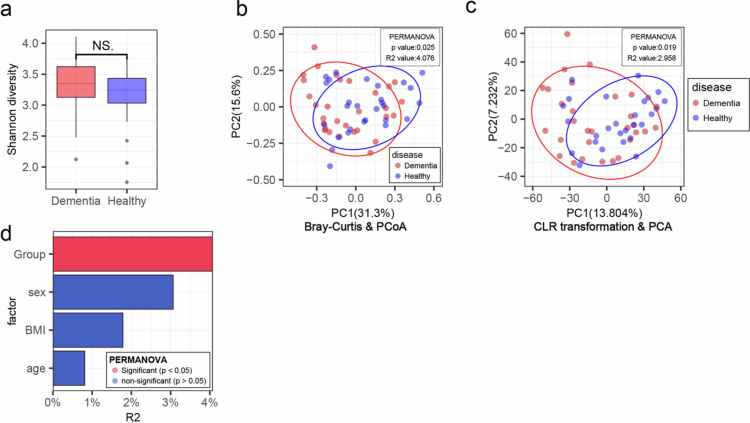
Compositional differences in the oral microbiota of dementia patients. (a) Boxplot comparing Shannon diversity between dementia and healthy controls. A two-tailed Mann‒Whitney U test was used to evaluate statistical significance (NS; no significance). Boxplot elements: median (center line), interquartile range (box edges at 25th and 75th percentiles), and whiskers extending to 1.5× the interquartile range. (b) Principal coordinate analysis (PCoA) illustrating compositional differences in the microbiota between groups, with PERMANOVA results shown in the upper-right corner. (c) Principal component analysis (PCA) of centered log-ratio (CLR)- transformed relative abundances using the Aitchison distance, illustrating compositional differences in the microbiota between groups; PERMANOVA results are shown in the upper-right corner. (d) Bar plot displaying PERMANOVA R values for each variable; red bars indicate variables with statistically significant effects. PCoA, principal coordinate analysis; PERMANOVA, permutational multivariate analysis of variance.

Although alpha diversity was not significantly different between the dementia and healthy control groups, with only a slight increase in diversity indices in the dementia group (Figure 1a**,** Supplementary Figure 1a-b), PCoA based on Bray‒Curtis distances revealed a significant difference in microbial composition between the two groups (Figure 1b). To account for the compositional nature of microbiome data, we additionally performed PCA using Aitchison distance on CLR-transformed relative abundances to validate the beta-diversity results; the findings were consistent with the PCoA analysis (Figure 1c). Furthermore, permutational multivariate analysis of variance (PERMANOVA) revealed that dementia status was the only variable significantly associated with the microbial profile, whereas sex, BMI, and age had no significant associations (Figure 1d). These analyses indicate that dementia status is associated with a distinct oral microbiome composition, independent of age, sex, and BMI.

### Oral bacterial species associated with dementia

**Figure 2.**
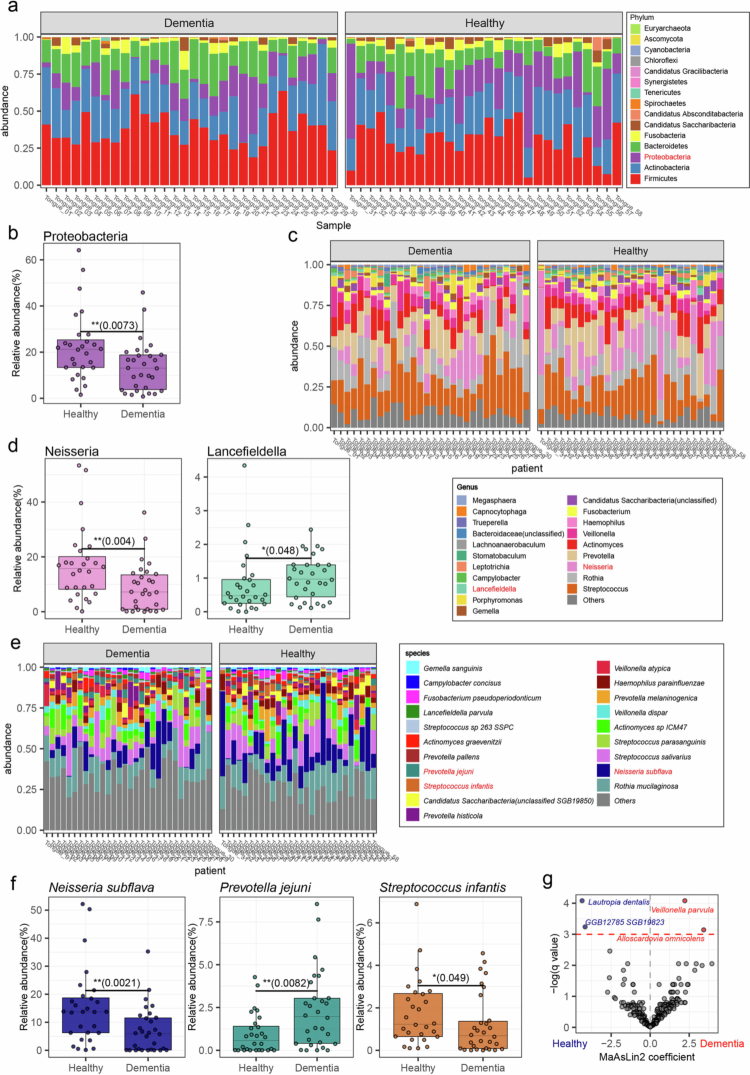
Differentially abundant oral microbiota between dementia and healthy control groups. (a) Bar plot of phylum-level relative abundance of oral microbiota in dementia patients and healthy controls. Red-labelled Phyla indicate significantly differentially abundant clades. (b) Boxplot of Proteobacteria relative abundance between groups; significance determined by a two-tailed Mann–Whitney U test (**; *p* < 0.01). (c) Bar plot of genus-level relative abundance of oral microbiota. Red labels indicate significantly differentially abundant genera. (d) Boxplot showing relative abundance of *Neisseria* and *Lancefieldella* between groups; significance determined by a two-tailed Mann–Whitney U test (**; *p* < 0.01, *; *p* < 0.05). (e) Bar plot of species-level relative abundance of oral microbiota. Rel-labelled species are significantly differentially abundant. (f) Boxplot comparing the relative abundance of three species between groups; significance determined by a two-tailed Mann–Whitney U test (**; *p* < 0.01, *; *p* < 0.05). (g) Differential species-level analysis adjusted for covariates using MaAsLin2. Each point represents a species, with red indicating enrichment in dementia group and blue indicating enrichment in healthy control group (*q*-value < 0.05). Boxplot elements: median (center line), interquartile range (box edges at 25th and 75th percentiles), and whiskers extending to 1.5× the interquartile range.

From the microbiomes of the tongue-coating samples, we identified a total of 15 phyla, 52 classes, 66 orders, 91 families, 173 genera, and 427 species. At the phylum level, Firmicutes, Actinobacteria, Proteobacteria, and Bacteroidetes were the dominant phyla in both groups. Among them, Firmicutes was the most dominant phylum, followed by Actinobacteria, in both groups. Proteobacteria were significantly more abundant in the healthy control group (median 22.25%) than in the dementia group (median 13.59%) (*p*-value = 0.0073) (Figure 2a-b**,** Supplementary Table 2). At the genus level, *Streptococcus, Rothia, Prevotella*, and *Neisseria* were the dominant genera (Table 1). *Neisseria* was significantly more abundant in the healthy control group and *Lancefieldella* was more abundant in the dementia group (Figure 2c-d**,** Table 1).

**Table 1.** Comparison of relative abundance at genus level.

	Dementia	Healthy control	*p-*value
*Streptococcus*	22.305	18.693	0.3257
*Rothia*	13.468	13.780	0.7051
*Neisseria*	8.701	16.930	0.0040*
*Prevotella*	12.534	10.980	0.1602
*Actinomyces*	8.605	8.913	0.7399
*Veillonella*	7.082	5.264	0.1112
*Haemophilus*	3.262	3.795	0.1901
*Porphyromonas*	2.544	1.965	0.8470
*Candidatus Saccharibacteria_unclassified*	1.617	2.471	0.1330
*Fusobacterium*	1.925	1.845	0.7051
*Leptotrichia*	1.450	1.230	1.0000
*Gemella*	1.327	0.948	0.4985
*Lancefieldella*	1.016	0.799	0.0485*
*Bacteroidaceae_unclassified*	0.825	0.905	0.9876
*Campylobacter*	0.770	0.833	0.5113
*Stomatobaculum*	0.830	0.729	0.5860
*Capnocytophaga*	0.808	0.682	0.4349
*Lachnospiraceae_unclassified*	0.809	0.616	0.9430
*Lachnoanaerobaculum*	0.489	0.591	0.4051
*Megasphaera*	0.526	0.535	0.4272
*Other*	9.105	7.494	0.2421

Median relative abundances (%) of the top 20 most abundant oral microbiota genera in dementia and healthy control groups. A two-tailed Mann‒Whitney U test was used to evaluate statistical significance.

^*^ *p* < 0.05.

At the species level, *Rothia mucilaginosa*, *Streptococcus salivarius*, *Neisseria subflava*, and *Streptococcus parasanguinis* were the dominant species. *Neisseria subflava* was significantly more abundant in the healthy control group (median 15.43%) than in the dementia group (median 7.19%) (*p*-value = 0.0021). *Prevotella jejuni*, although not dominant, was more abundant in the dementia group (2.31%) than in the healthy control group (median 0.95%) (*p*-value = 0.0082). Similarly, *Streptococcus infantis* was more abundant in the healthy control group (1.78%) than in the dementia group (median 1.20%) (*p*-value = 0.0490) (Figure 2e-f**,** Table 2).

**Table 2.** Comparison of relative abundance at species level.

	Dementia	Healthy control	*p-*value
*Veillonella dispar*	3.086	2.757	0.5623
*Veillonella atypica*	2.874	1.906	0.1043
*Streptococcus* sp. *A12*	1.246	0.778	0.3039
*Streptococcus salivarius*	8.947	8.199	0.7989
*Streptococcus parasanguinis*	6.781	4.971	0.1797
*Streptococcus infantis*	1.205	1.783	0.0490*
*Rothia mucilaginosa*	12.187	13.011	0.4533
*Prevotella pallens*	1.328	1.470	0.9318
*Prevotella melaninogenica*	2.914	3.967	0.3007
*Prevotella jejuni*	2.305	0.952	0.0082*
*Prevotella histicola*	2.885	2.276	0.4363
*Porphyromonas pasteri*	1.367	1.503	0.1990
*Neisseria subflava*	7.193	15.431	0.0021*
*Lancefieldella parvula*	0.961	0.791	0.0885
*Haemophilus parainfluenzae*	2.986	3.732	0.0800
*Fusobacterium pseudoperiodonticum*	1.133	1.466	0.3382
*Candidatus Saccharibacteria unclassified SGB19850*	1.387	2.209	0.1188
*Actinomyces* sp. *ICM58*	1.050	1.023	0.6508
*Actinomyces* sp. *ICM47*	4.327	5.047	0.3533
*Actinomyces graevenitzii*	1.492	1.452	0.8824

Median relative abundances (%) of the top 20 most abundant oral microbiota species in dementia and healthy control groups. A two-tailed Mann‒Whitney U test was used to evaluate statistical significance.

^*^ *p* < 0.05.

To account for potential confounders and accurately detect species enriched in the dementia group, we performed differential abundance analysis using MaAsLin2 [45]. This analysis revealed that *Veillonella parvula* (*q*-value = 0.0169) was the most significantly enriched species in the dementia group, followed by *Alloscardovia omnicolens* (*q*-value = 0.0432) (Figure 2g**,** Table 3). In addition, association of these species with dementia was confirmed using MaAsLin2 analysis with CLR transformation (Supplementary Figure 1c). These results suggest that *V. parvula* and *A. omnicolens* may serve as noninvasive oral metagenomic markers of dementia.

**Table 3.** MaAsLin2 analysis of differentially abundant species between Dementia and Healthy control groups.

Species	Coefficient	*q*-value
*Lautropia dentalis*	−4.3590	0.0169*
*Veillonella parvula*	2.2200	0.0169*
*GGB12785 SGB19823*	−4.1657	0.0393*
*Alloscardovia omnicolens*	3.4293	0.0432*
*Neisseria subflava*	−2.5825	0.0856
*Alloprevotella tannerae*	2.3119	0.1286
*Bergeyella cardium*	−1.4379	0.1286
*Bifidobacterium dentium*	3.9523	0.1286
*Olsenella uli*	2.9477	0.1286
*Streptococcus gordonii*	1.8179	0.1286
*Prevotella jejuni*	3.3257	0.1364
*GGB1843 SGB2524*	−1.7139	0.1589
*Lancefieldella rimae*	2.8563	0.1589
*Neisseria elongata*	−2.6107	0.2023
*Prevotella buccae*	1.8412	0.2064
*Streptococcus infantis*	−1.6455	0.2125

Differential abundance analysis using MaAsLin2 identified microbial species that differed between Dementia and healthy control groups. Positive coefficients indicate higher abundance in dementia group, whereas negative values indicate higher abundance in healthy control group.

^*^ *q*-value < 0.05.

In contrast, *Lautropia dentalis* (*q*-value = 0.0169) was the most significantly enriched species in the healthy control group, followed by GGB12785_SGB19823, an uncharacterized species from the family Candidatus Nanogingivalaceae within the Candidate Phyla Radiation (CPR) group (*q*-value = 0.0393) (Figure 2g**,** Table 3). These taxa, which were negatively associated with dementia status, may enable a more reliable diagnosis.

### Oral bacterial metabolic pathways linked to dementia

**Figure 3.**
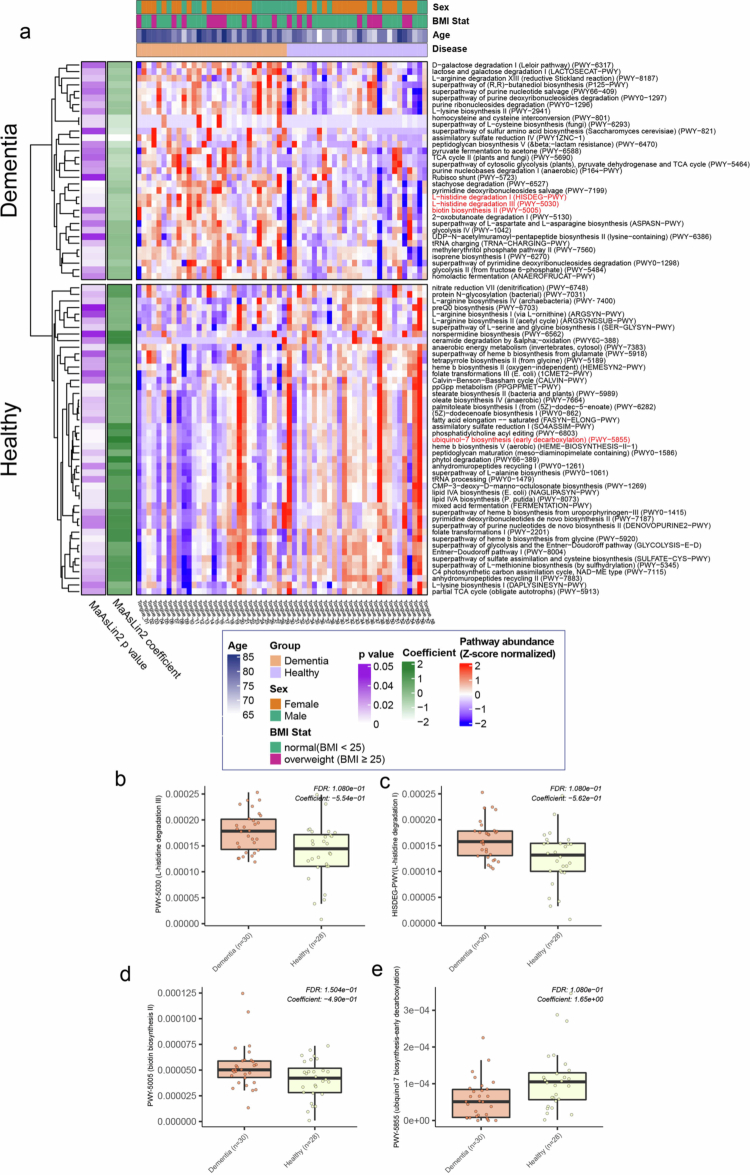
Comparison of metabolic pathway abundance between dementia and healthy controls. (a) Heatmap of differentially enriched metabolic pathways of the oral microbiome. Feature values were normalized using row-wise Z score transformation (mean = 0, standard deviation = 1) to emphasize relative differences across samples. Rows represent pathways significantly enriched in either group (MaAsLin2 *p*-value < 0.05); columns represent individual samples with annotated metadata shown above. (b-e**)** Boxplots of relative abundance of selected metabolic pathways: (b) L-histidine degradation III; (c) L-histidine degradation I; (d) biotin biosynthesis II; and (e), ubiquinol-7 biosynthesis (early decarboxylation).

To further explore oral bacterial metabolic functions associated with dementia, we conducted pathway-level differential abundance analysis using MaAsLin2 (Figure 3a). Among the microbial metabolic pathways, L-histidine degradation III (PWY-5030; *p*-value = 0.0055, false discovery rate [FDR] = 1.08e-01), L-histidine degradation I (HISDEG-PWY; *p*-value = 0.0049, FDR = 1.08e-01), and biotin biosynthesis II (PWY-5005; *p*-value = 0.0327, FDR = 1.50e-01) was enrichment in the dementia group relative to the healthy control group (Figure 3b–d). In contrast, the ubiquinol-7 biosynthesis pathway (early decarboxylation) (PWY-5855; *p*-value = 0.0022, FDR = 1.08e-01) was enriched in the healthy control group (Figure 3e).

Overall, these findings highlight possible links between metabolic disruptions in the oral microbiome and the pathophysiology of neurodegenerative conditions.

### Oral bacterial species correlated with dementia severity

**Figure 4.**
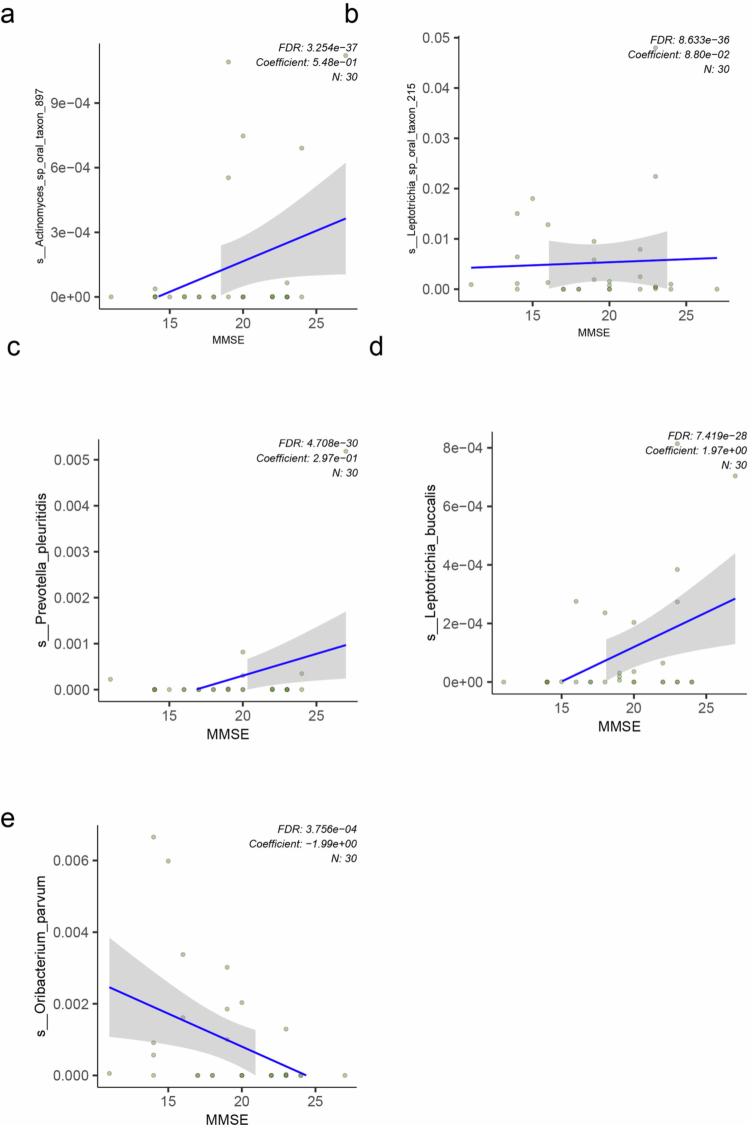
Oral bacterial species associated with K-MMSE scores in dementia patients. (a–e) Scatterplots of species significantly associated with K-MMSE scores, as identified using MaAsLin2 (*q* < 0.05). Each plot shows positive or negative correlation between relative abundance and cognitive function: (a) *Actinomyces* sp. *oral taxon 897*; (b) *Leptotrichia* sp. *oral taxon 215*; (c) *Prevotella pleuritidis*; (d) *Leptotrichia buccalis*; and (e) *Oribacterium parvum*. K-MMSE-Korean Mini-Mental State Examination, 2nd edition.

To identify microbial species associated with dementia severity, we performed MaAsLin2 regression using K-MMSE scores, which assess on orientation, memory, and language. The abundance of *Prevotella pleuritidis* was positively correlated the K-MMSE score, with the abundance increasing alongside higher scores (FDR = 4.708e-30, coefficient = 0.297) (Figure 4a). Similarly, *Actinomyces* sp. *oral taxon 897* (FDR = 3.254e-37, coefficient = 0.548), *Leptotrichia buccalis* (FDR = 7.419e-28, coefficient = 1.97), and *Leptotrichia* sp. *oral taxon 215* (FDR = 8.633e-36, coefficient = 8.80e-02) also demonstrated significantly increased abundances with higher K-MMSE scores (Figure 4b-d). Higher K-MMSE scores indicate less severe dementia. Therefore, oral microbial species that are positively correlated with K-MMSE score may be associated with relatively better cognitive status in dementia patients. In contrast, *Oribacterium parvum* abundance was negatively correlated with the K-MMSE score, with its relative abundance decreasing as the K-MMSE score increased (FDR = 3.756e-04, coefficient = –1.99e+00) (Figure 4e), suggesting an association with worse cognitive status.

### Oral bacterial metabolic pathway activities correlation with dementia severity

**Figure 5.**
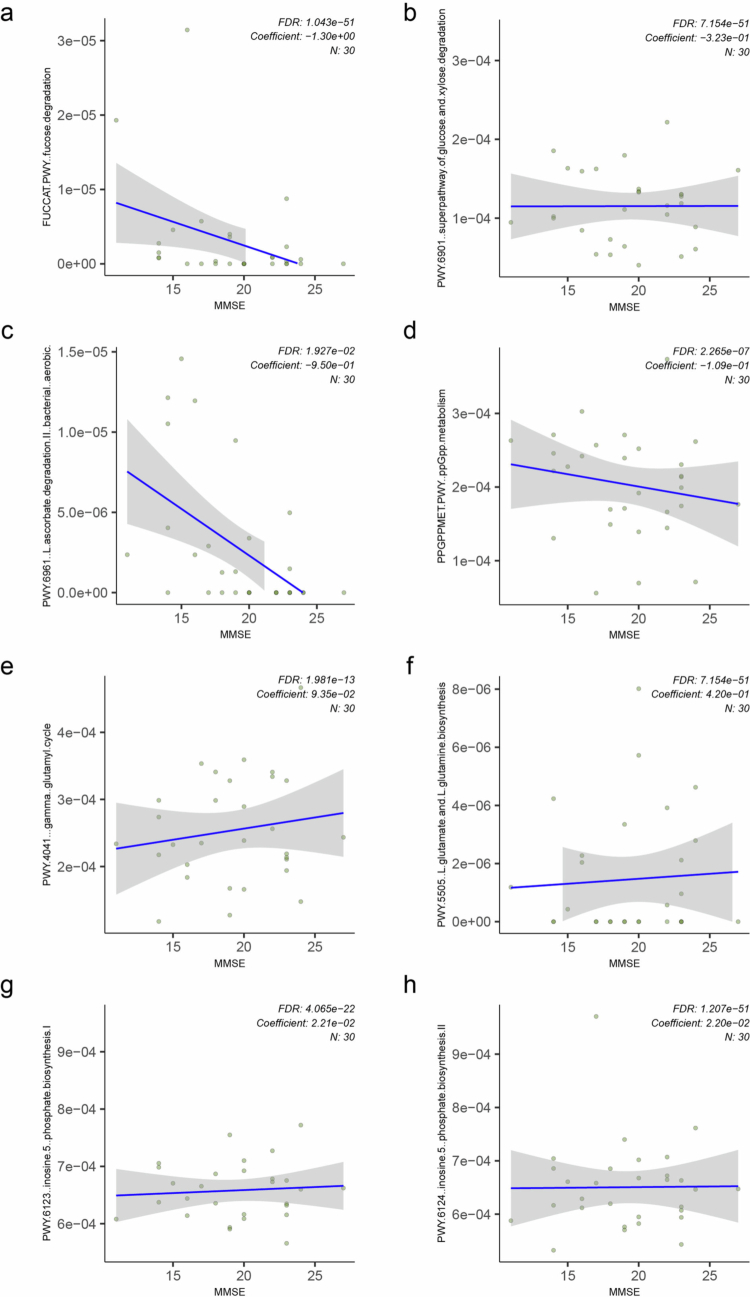
Oral bacterial metabolic pathways correlated with K-MMSE scores in dementia group. **(**a–h) Scatterplots showing metabolic pathways significantly associated with K-MMSE scores, as identified using MaAsLin2 (*q* < 0.05). Pathways included: (a) Fucose degradation; (b) Superpathway of glucose and xylose degradation; (c) L-ascorbate degradation II (bacterial, aerobic); (d) ppGpp metabolism; (e) Gamma‒glutamyl cycle; (f) L-glutamate and L-glutamine biosynthesis; (g) Inosine 5'-phosphate biosynthesis I; and (h) Inosine 5'-phosphate biosynthesis II. K-MMSE-Korean Mini-Mental State Examination, 2nd edition.

Using a similar MaAsLin2 regression analysis, we found that the abundance of several bacterial metabolic pathways was negatively correlated with K-MMSE scores in dementia patients. These pathways included fucose degradation (FUCCAT-PWY), the superpathway of glucose and xylose degradation (PWY-6901), L-ascorbate degradation II (PWY-6961), and ppGpp metabolism (PPGPPMET-PWY) (Figure 5a–d). Collectively, these findings suggest that greater dementia severity is associated with coordinated alterations in carbohydrate/energy metabolism and stress-response pathways in the oral microbiome.

We also identified several bacterial metabolic pathways whose abundance was positively correlated with K-MMSE scores, including the gamma-glutamyl cycle (PWY-4041), L-glutamate and L-glutamine biosynthesis (PWY-5505), and both inosine 5'-phosphate biosynthesis I (PWY-6123) and II (PWY-6124), reflecting increased abundance with better cognitive function (Figure 5e–h). Together, these observations raise the possibility that glutamine/glutamate-related metabolic functions encoded by oral commensals are associated with dementia severity and may contribute to host neurophysiology.

## Discussion

This study compared the oral microbiomes of older adults with dementia and cognitively healthy controls by analyzing tongue-coating samples via shotgun metagenomic sequencing. We utilized shotgun metagenomic sequencing, which offers higher taxonomic and functional resolution than 16S rRNA-based evaluation of oral microbiome profiles in dementia patients. Additionally, tongue-coating samples have practical advantages owing to their relative stability and ease of collection, making them potentially valuable, non-invasive biomarker sources for disease monitoring.

Although there were no statistically significant differences in alpha diversity, beta diversity analyses indicated a clear separation in overall microbiota compositions between dementia patients and healthy controls. Whinin this community-level shift, *V. parvula* and *A. omnicolens* were significantly enriched in the dementia group, suggesting their potential as key oral microbial biomarkers associated with dementia.

Notably, *V. parvula* is often considered a constituent of clinically healthy oral microbiota and has been reported to be more abundant in individuals with healthy periodontal status [46]. However, accumulating evidence suggests that *V. parvula* may also be linked to neuroinflammation and neurodegenerative diseases, including dementia [11,47,48]. Mechanistically, lipopolysaccharide (LPS) from *V. parvula* has been proposed to increase barrier permeability and activate macrophages via TLR4 pathway [49], thereby promoting proinflammatory cytokine release and oxidative stress, processes implicated in neurodegenerative pathology. Consistently, *V. parvula* exacerbated neuroinflammation in a PD mouse model by enhancing infiltration of IFN-*γ*-producing T-helper 1 (Th1) cells into the brain [47]. Collectively, these findings raise the possibility that oral *V. parvula* may contribute to dementia by promoting systemic inflammation and neuroinflammatory responses at distant sites.

Similarly, *A. omnicolens* has been reported to be more abundant in patients with post-stroke cognitive impairment than in stroke patients without cognitive impairment and healthy controls [39], supporting for its potential involvement in cognitive decline and neurodegenerative processes.

We also identified oral bacterial metabolic pathways associated with dementia based on inferred metagenomic functional potential. Specifically, genes assigned to histidine degradation and biotin biosynthesis pathways were enriched in the dementia group, whereas genes involved in ubiquinone/ubiquinol (coenzyme Q) biosynthesis were relatively enriched in healthy controls.

Histidine is important for neural function and has been implicated in antioxidant defense and modulation of oxidative stress. Consistent with this, altered histidine metabolism has been associated with neurodegeneration and AD progression [50,51]. In this context, the enrichment of microbial histidine degradation–related genes in dementia may reflect a microbiome shift linked to disturbed histidine availability or utilization, aligning with prior observations connecting histidine metabolism to neurodegenerative pathology.

In contrast, ubiquinone/ubiquinol (coenzyme Q) is a key component of mitochondrial electron transport and redox homeostasis, processes that are frequently disrupted in AD and related dementias [52]. Thus, the relative depletion of ubiquinone/ubiquinol biosynthesis genes in the dementia-associated microbiome may be consistent with broader mitochondrial and redox dysregulation observed in neurodegeneration.

Additionally, we found that the abundances of several bacterial species and metabolic pathways were significantly correlated with dementia severity, as measured by K-MMSE scores, in dementia patients. Because higher K-MMSE scores indicate less severe cognitive impairment, taxa or pathways positively correlated with K-MMSE may be associated with relatively preserved cognitive function, whereas those negatively correlated with K-MMSE may track with greater severity and potentially reflect processes linked to cognitive decline. Accordingly, oral microbial features correlated with K-MMSE could provide a simple, noninvasive readout for predicting dementia severity and cognitive status.

At the pathway level, fucose, glucose, and xylose degradation, together with L-ascorbate degradation and ppGpp metabolism, showed negative correlation with K-MMSE score, indicating relative enrichment of these functions with increasing dementia severity. Given that fucose, glucose, and xylose degradation constitute core carbohydrate catabolic routes, these results suggest increased utilization of host- and diet-derived sugars as cognitive impairment worsens. The negative association of L-ascorbate degradation with K-MMSE further implicates altered ascorbate handling, consistent with perturbed redox balance and oxidative stress responses [53]. In addition, ppGpp metabolism is indicative of activation of the bacterial stringent response and stress-related metabolic reprogramming [54].

Conversely, the gamma-glutamyl cycle, glutamate/glutamine biosynthesis, and inosine 5′-phosphate biosynthesis were positively correlated with K-MMSE scores, linking relatively preserved cognition to enrichment of these pathways. Notably, pathways related to glutamine/glutamate metabolism were among those associated with K-MMSE scores, highlighting a link to neurotransmitter-relevant metabolic functions. In line with this concept, an elevated gut microbial GABA/glutamate ratio has been proposed as a metabolic marker in mild autism spectrum disorder (ASD) [55]. By analogy, an imbalance in the oral microbial GABA/glutamate ratio may be associated with dementia severity; this hypothesis warrants future validation using targeted metabolomic measurements and longitudinal cohorts.

Mechanistically, glutamate is the major excitatory neurotransmitter and serves as the direct precursor for *γ*-aminobutyric acid (GABA), the major inhibitory neurotransmitter, while glutamine functions as an upstream reservoir supporting neuron–astrocyte metabolic coupling through the glutamate–glutamine cycle, a pathway implicated in brain development and neurodegenerative disorders [56]. Consistent with the relevance of microbial glutamate/glutamine metabolism to neurodegeneration, a recent large-cohort study of PD reported depletion of microbial genes involved in glutamate synthesis in gut metagenome [57].

It is essential in oral microbiome study design to carefully consider the sampling site. To minimize the influence of periodontitis- and caries-associated bacteria, the tongue dorsum was chosen for this study as it is less directly influenced by localized periodontal or carious lesions, where specific taxa such as *Porphyromonas gingivalis*, *Tannerella forsythia*, and *Streptococcus mutans* are more commonly found. Studies have shown that while taxa typically associated with dental caries and periodontitis are present in the tongue microbiota, they represent minority members (mean relative abundance < 0.1%) and do not form cohabiting commensal communities. Furthermore, the tongue-coating microbiota exhibits relative stability due to a moderate rate of biofilm shedding from tongue epithelial cells and associated microorganisms, making it a suitable site for microbiome studies [29,31].

Our study has several limitations. First, confounding factors remain despite strict patient selection criteria, rigorous metadata collection, and quality control of metagenomic sequencing reads to ensure accurate disease–microbiome associations. Patient-related variables (e.g. medication use, immune status, diet, and oral hygiene) and technical factors (e.g. sampling methods, sequencing platforms, and bioinformatic pipelines) may influence the oral microbiome, introducing inconsistencies across studies and complicating the identification of reliable microbial biomarkers. In particular, age, an established confounding factor that shapes the human oral microbiome, differed significantly between the dementia and healthy groups, with dementia cases being older, which may have influenced the observed associations [58]. To reduce false discoveries, we adjusted for age, sex, and BMI using PERMANOVA analysis and MaAsLin2, which demonstrated that dementia status was the only variable significantly associated with global microbiome structure. In addition, we were unable to comprehensively adjust for other potential confounders (e.g. socio-economic status, lifestyle factors, and health related variables) due to the limited sample size. Second, we did not stratify participants by dementia subtypes, such as AD, PD, or vascular dementia, which limited our ability to examine potential compositional differences between subtypes and may have introduced additional heterogeneity in microbiome profiling.

Future studies should include larger participant groups with more detailed covariate data. Furthermore, standardized methodologies and longitudinal designs should be applied to clarify the mechanism underlying the causal relationship between the oral microbiome and dementia progression.

## Conclusions

Shotgun metagenomic analysis of tongue-coating samples revealed distinct differences in microbial composition and metabolic function between dementia patients and cognitively healthy older adults. *V. parvula* was significantly enriched in the dementia group, while *L. dentalis* was more abundant in the healthy group. Functionally, histidine degradation and biotin biosynthesis pathways were more abundant in dementia patients, whereas ubiquinol biosynthesis was enriched in healthy individuals. Furthermore, several microbial taxa and pathways showed significant correlations with K-MMSE scores, highlighting their potential as noninvasive oral biomarkers associated with cognitive decline.

## Supplementary Material

Supplementary_Figure1.jpg Supplementary_Figure1.jpg

Cha_Jeong_etal_SupplementaryInfo_20260131.pdf Cha_Jeong_etal_SupplementaryInfo_20260131.pdf

## Data Availability

The data supporting this study are available from the corresponding author, Dr. Bock-Young Jung, upon reasonable request.
