# Supplementary figures and images for: Shotgun metagenomic analysis of the tongue-coating microbiome reveals oral microbes and their functions in older adults with dementia
Source: J Oral Microbiol. 2026 Mar 11;18(1):2643036. doi: 10.1080/20002297.2026.2643036 (PMC12981268; doi:10.1080/20002297.2026.2643036)

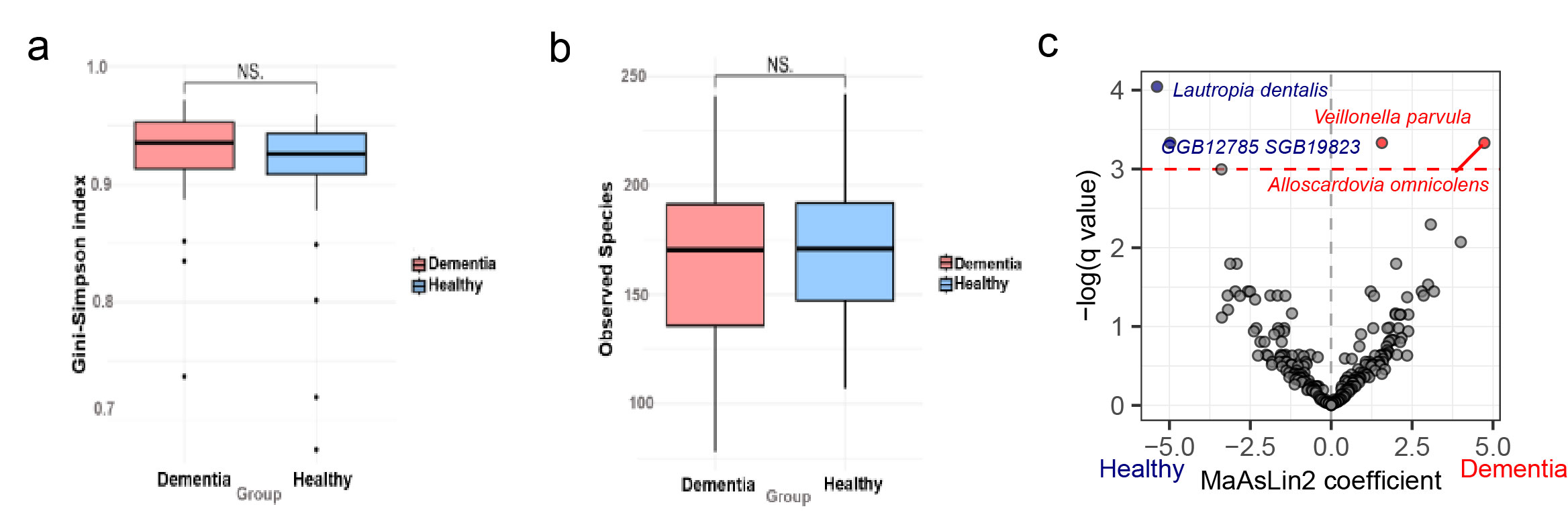

Supplement: Supplementary_Figure1.jpg [file ZJOM_A_2643036_SM3815.jpg]
