# Supplementary material for: Shotgun metagenomic analysis of the tongue-coating microbiome reveals oral microbes and their functions in older adults with dementia
Source: J Oral Microbiol. 2026 Mar 11;18(1):2643036. doi: 10.1080/20002297.2026.2643036 (PMC12981268; doi:10.1080/20002297.2026.2643036)
Supplement: Cha_Jeong_etal_SupplementaryInfo_20260131.pdf [file ZJOM_A_2643036_SM3814.pdf]

Supplementary Information

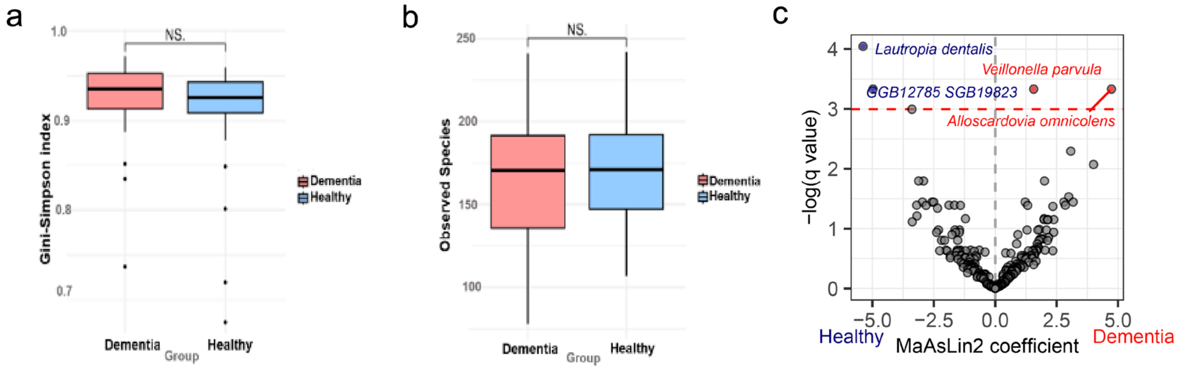

Supplementary Fig. 1 | Alpha diversity analysis of the oral microbiome in dementia and healthy control groups. Boxplot comparing alpha diversity between dementia patients and healthy controls. **a,** Gini–Simpson index and **b,** number of observed species. A two-tailed Mann–Whitney U test was used to evaluate statistical significance (NS; no significance). Boxplot elements: median (center line), interquartile range (box edges at 25th and 75th percentiles), and whiskers extending to 1.5× the interquartile range. **c,** Covariate-adjusted species-level differential analysis using MaAsLin2 after CLR transformation. Each point represents a species, with red indicating enrichment in the dementia group and blue indicating enrichment in the healthy control group ($q <$ 0.05).

Supplementary Table 1 | Demographic and clinical characteristics of participants.

	**All (n = 58)**	**Dementia (n = 30)**	**Healthy control (n = 28)**	***p* value**
**Male, n (%)**	24 (41.38)	14 (46.67)	10 (35.71)	0.5622^{*}
**Female, n (%)**	34 (58.62)	16 (53.33)	18 (64.29)	
**Age, mean (SD)**	77.48 (4.99)	79.60 (4.17)	75.21 (4.86)	$0.0005^{\dagger}$
**K-MMSE, mean (SD)**	23.64 (5.42)	19.23 (3.86)	28.36 (1.22)	$< 0.0001^{\ddagger}$
**BMI, mean (SD)**	23.57 (2.84)	23.93 (3.18)	23.18 (2.42)	$0.3099^{\dagger}$

K-MMSE, Korean Mini-Mental State Examination 2nd edition; SD, standard deviation; BMI, body mass index. Group comparisons were performed using the chi-square test (*; $p < 0.05$), Student's t test (†; $p < 0.05$), or two-tailed Mann–Whitney U test (‡; $p < 0.05$).

Supplementary Table 2 | Comparison of relative abundance at phylum level.

	Dementia (%)	Healthy control (%)	p value†
Actinobacteria	25.221	24.918	0.9446
Ascomycota	0.001	0	0.3515
Bacteroidetes	17.910	15.212	0.0583
Candidatus Absconditabacteria	0.082	0.495	0.1051
Candidatus Gracilibacteria	0.006	0.019	0.2266
Candidatus Saccharibacteria	1.814	2.798	0.0626
Chloroflexi	0.003	0.001<	0.2828
Cyanobacteria	0.001	0	0.1759
Euryarchaeota	0.001<	0.001<	1.0000
Firmicutes	37.308	30.309	0.0915
Fusobacteria	3.667	3.744	0.6265
Proteobacteria	13.591	22.254	0.0073*
Spirochaetes	0.212	0.143	0.7556
Synergistetes	0.051	0.015	0.6771
Tenericutes	0.133	0.093	0.8033

Median relative abundances (%) of oral microbiota at the phylum level in dementia and healthy control groups. A two-tailed Mann–Whitney U test was used to evaluate statistical significance (*; $p < 0.05$) $^{*}p < 0.05$
